# Medical Organization

**Published:** 1882-02

**Authors:** 


### MEDICAL ORGANIZATION.

Individual effort, no matter how well directed, how intelligently prosecuted or how perseveringly continued, often fails. Fails, not on account of an unworthy object, or of inefficient management or of defective ability; but from weakness—a relative weakness of the individual—a weakness out of proportion to the forces which, ofttimes of necessity, he must oppose.

Strength lies in union. Than this, no principle is better understood or more generally acknowledged. In the maintenance of governments, in the conduct of great enterprises, and in the operation of the ordinary business affairs of life this doctrine receives abundant confirmation.

The strength begot of combination is not reckoned merely by the sum of the individual power exercised in concert, but the collateral influences which a judicious organization can exert may be enormous, and, like the power of the lever or the pulley, may accomplish incredible results, almost without the consciousness of effort upon the part of the component factors of such an organization.

The aggregation of the total strength, direct and consequential, of an effectively organized body of intelligent men, it matters not in what particular field their energies are expended, may be such as to make itself felt throughout a community, throughout a whole country or even throughout the world.

The extent to which this element of power is ignored by medical men is painfully apparent. It is safe to assert that no part of any community stands higher in public estimation than the physicians of that community, regarded as individuals. No man exercises more absolute power, receives more implicit obedience, is depended upon with more child-like confidence, or is held in more affectionate esteem than the physician; and yet, as a class, how pitifully feeble is their influence! Collectively, how impotent and powerless they appear as compared with many other classes of men possessing far inferior qualifications.

In view of these indisputable facts it is pertinent and profitable to inquire into the cause of this unfortunate and undesirable state of things, and to propose, if possible, a remedy. To our mind it is clear that the whole trouble is traceable to a want of professional unity. It is division, dissension, and discord within the ranks that enervates, depresses and cripples.

It is true that the presence within the profession of many incapable and unworthy men tends to lower its standing and to lessen its influence as a whole, but this defect is common to other callings, and could be easily counteracted if harmony and unity prevailed among the better and more influential members, who could, if they would, fix the gauge, which a willing public would gladly accept.

In a word, then, organization is the remedy. A combination of the interests and a combination of the available strength of the reputable medical men of any community would promptly place the profession, which we claim to love and honor, in a totally different and a far better relation toward the public, and gradually but surely secure for it that position in public confidence which it richly deserves and can justly claim.

But it is not alone this general good that can be obtained and fostered by effective organization. There is a personal advantage of no mean account. Every practitioner of medicine must feel that his lot, so far as his daily round of professional work is concerned, is a somewhat isolated one. Now, this isolation, with the habits of thought which it is liable to engender, is best relieved by the associations which an active, well-organized medical society alone can supply—by the corrective influences which are here most pleasantly encountered. It will be a bright day in the history of the profession when every county in this State, and in all the States, has its medical organization, and when our state associations number among their members every respectable medical man throughout the length and breadth of the country. Then the profession will exert an influence commensurate with its deserts, and physicians will become, in the fullest sense, mutually helpful and mutually helped.

